# Prevalence of immunization against influenza in elderly Brazilians: National Health Survey, 2019

**DOI:** 10.1590/1806-9282.20230790

**Published:** 2024-03-04

**Authors:** Claudio José dos Santos, Luiz Marques Campelo, Elaine Cristina Torres Oliveira

**Affiliations:** 1 Universidade Federal da Bahia, Institute of Public Health – Salvador (BA), Brazil.; 2 Universidade de São Paulo, Faculty of Public Health – São Paulo (SP), Brazil.; 3 Ministry of Health – Brasília (DF), Brazil.; 4 Universidade Estadual de Ciências da Saúde de Alagoas – Maceió (AL), Brazil.

**Keywords:** Influenza vaccines, Aged, Health surveys, Brazil

## Abstract

**OBJECTIVE:**

The aim of this study was to estimate the prevalence of influenza immunization in elderly people in Brazil in 2019.

**METHODS:**

This is a population-based cross-sectional study. The Brazilian individuals (≥60 years) who participated in the 2019 National Health Survey were included. The survey was conducted in permanent households in Brazil from August 2019 to March 2020. The prevalences of influenza vaccination and their respective confidence intervals (95%CI) were estimated according to sociodemographic characteristics and the diagnosis of chronic diseases.

**RESULTS:**

The prevalence of influenza vaccination was 72.4% (95%CI 71.5–73.2), with statistically significant differences observed between genders (p=0.001), age groups (p=0.001), and those living with a spouse/partner (p=0.002). Significant differences were found in groups with arterial hypertension (75.2%, p<0.001), diabetes (77.2%, p<0.001), and arthritis or rheumatism (75.5%, p<0.001).

**CONCLUSION:**

A global prevalence of influenza vaccination of 72.4% was estimated among elderly people in Brazil.

## INTRODUCTION

Influenza, an acute viral infection caused by *Myxovirus influenzae*, remains one of the greatest public health challenges in the world^1,2^. Every year, almost 1 billion new cases of this disease are estimated worldwide, of which between 3 and 5 million are severe cases, resulting in 290,000 to 650,000 deaths from respiratory diseases related to flu and its complications^3^.

In Brazil, in 2020, 57,603 deaths were recorded by ICD-10 J09–J18, which includes deaths from influenza and primary viral or secondary bacterial pneumonia, resulting in a mortality rate of 19.7 deaths per 10,000 elderly people for the studied causes^4^.

Given this scenario, the World Health Organization recommends annual vaccination against influenza as the most effective strategy to reduce hospital admissions, costs with supplies for treating secondary conditions, and the risk of death from complications related to influenza^5^.

In Brazil, annual immunization campaigns against the influenza virus have been carried out since 1999 for groups considered a priority by the Ministry of Health, including the elderly population^6,7^.

Despite the fact that the trivalent “flu” vaccine is available through the National Immunization Program, free of charge, and widely available in vaccination units distributed across the country for such groups, studies conducted nationally have been pointing to a downward trend in coverage of this immunogen in the Brazilian population aged 60 years or older^8^, lower adherence of specific subgroups of elderly people to this prevention strategy^9^, in addition to the failure to reach national and international coverage targets^10^. A study conducted in 2020 in 133 sentinel cities across Brazilian states (the most populous cities) revealed a vaccination coverage for influenza among elderly people of approximately 83% (the recommended target being 90%)^11^. This makes constant monitoring necessary for adherence rates to annual influenza vaccination in this population and associated factors.

In this sense, the present study aimed to estimate the prevalence of influenza immunization in elderly people in Brazil in 2019.

## METHODS

### Study type

This is a population-based cross-sectional study using publicly available data from elderly people (≥ 60 years) who participated in the PNS 2019, a national household survey conducted in Brazil.

### Context

The PNS is the most comprehensive survey of health and provides information on the determining factors, conditions, and health needs of the population. It includes individuals living in permanent private households, covering both urban and rural areas. The 2019 edition was conducted in partnership between the Brazilian Institute of Geography and Statistics (IBGE) and the Ministry of Health of Brazil. This survey provides a comprehensive representation of the population living in private households across the five regions of the country. The survey was conducted from August 2019 to March 2020. Details regarding conceptual/methodological aspects and data collection for PNS 2019 are published^12^.

### Participants

**Figure 1:**
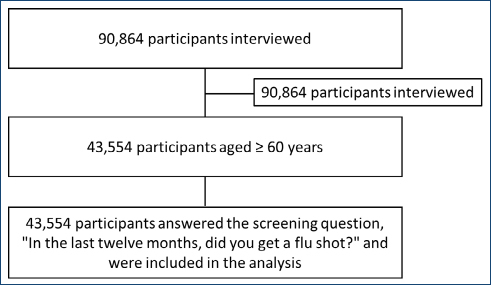
Sample selection process.

The study analyzed data from Brazilian participants aged 60 years or older who responded to the health questionnaire for individuals aged 60 years and over, included in the set of individual questions of the PNS. Sampling for PNS 2019 was done through a three-stage cluster sampling, with stratification of primary units (census sectors/cluster of sectors); households formed the units of the second stage, and one resident from each household formed the units of the third stage (both selected by simple random sampling). PNS 2019 selected 94,114 Brazilians, of whom 90,846 were interviewed. Among them, 43,554 were residents aged 60 years or older^12^. The PNS 2019 database records a total of 43,554 responses to the filter question for this research (Figure 1).

### Variables

The outcome variable was defined based on the following question: “In the last twelve months, have you received the influenza vaccine?” with available responses being yes or no. Independent variables were obtained from the “general characteristics and education of residents” and “chronic diseases” modules and included administrative region of residence (north, northeast, southeast, south, or midwest), gender (male or female), age group (60–69 years or 70 years or older), living with spouse/partner (yes or no), literacy (yes or no), race/ethnicity (white or yellow, black, brown, or indigenous), per capita monthly income (≤1 minimum wage or>1 minimum wage), and previous diagnosis (yes or no) of a noncommunicable chronic disease (hypertension, diabetes, heart disease, stroke, asthma, arthritis or rheumatism, cancer, chronic renal insufficiency, lung disease, or chronic obstructive pulmonary disease – COPD).

### Data source and measurement

The data from the PNS 2019 are publicly available and can be accessed on the IBGE website at the following link: https://www.ibge.gov.br/estatisticas/sociais/saude/9160-pesquisa-nacional-de-saude.html?=&t=microdados. This information was collected through electronic questionnaires conducted on mobile data collection devices. The questions were self-reported, and the answers were obtained through face-to-face interviews.

### Bias control

The PNS 2019 employed a random cluster sampling method in stages to minimize potential selection biases. To ensure the quality of the collected data, all those involved in data collection, supervision, and coordination underwent rigorous training to reduce information bias. Additionally, expansion factors and sample weights were applied in data analysis. This approach is justified due to the complex sample design and distinct selection probabilities allowed by the PNS.

### Study size

In planning the sample size for the PNS 2019, various factors were taken into account. This included estimating proportions with a desired level of precision expressed in 95% confidence intervals (95%CI), as well as the impact of the sampling plan. Furthermore, the number of households in each primary sampling unit was carefully selected, and the sample size was distributed according to population subgroups. The proportion of households containing individuals within the age range of interest was also considered in the sample calculation^12^.

### Statistical methods

Data were analyzed using the SPSS software version 25, in the Complex Samples module, which considers the effects of stratification and clustering in estimating indicators and their measures of precision. Prevalences of influenza vaccination and their respective 95%CIs were estimated overall and according to independent variables. Associations were verified using the chi-square test with a significance level of 5%.

### Ethical aspects

The PNS was approved by the Research Ethics Committee of the Ministry of Health under CAAE no. 11713319.7.0000.0008 and opinion no. 3.529.376.

## RESULTS

The data from 43,554 elderly people were analyzed, with 43.3% males (95%CI 42.7–43.8) and 56.7% females (95%CI 56.2–57.3). The age of the sample ranged from 60 to 112 years, with a mean of 70.1 years (95%CI 69.9–70.2). Distribution by age group revealed that 55.5% were between 60 and 69 years (95%CI 54.6–56.3) and 44.5% were 70 years or older (95%CI 43.7–45.4).

The prevalence of influenza vaccination was 72.4% (95%CI 71.5–73.2), with statistically significant differences observed between genders (p=0.001), age groups (p=0.001), and living with spouse/partner (p=0.002). However, there was no statistically significant difference in vaccination prevalence for the variables of literacy, race/color, region, and per capita income (p>0.05) (Table 1).

**Table 1:** Influenza vaccination among elderly Brazilians, according to sociodemographic characteristics.

Variables	Vaccinated			Not vaccinated			p-value
	No	%	95%CI	No	%	95%CI	
Region							
North	5067	72.0	70.2–73.7	2059	28.0	26.3–29.8	0.110
Northeast	10799	71.5	70.3–72.7	4384	28.5	27.3–29.7	
Southeast	7912	72.3	70.8–73.9	2743	27.7	26.1–29.2	
South	4363	72.7	71.0–74.3	1678	27.3	25.7–29.0	
Midwest	3406	75.6	73.5–77.5	1143	24.4	22.5–26.5	
Gender							
Male	13618	71.2	70.1–72.3	5585	28.8	27.7–29.9	**0.001**
Female	17929	73.2	72.3–74.2	6422	26.8	25.8–27.7	
Age group							
60–69 years	17017	70.3	69.2–71.3	7230	29.7	28.7–30.8	**0.000**
70 years or older	14530	75.0	73.8–76.2	4777	25.0	23.8–26.2	
Lives with spouse/partner							
Yes	18264	73.4	72.2–74.5	6553	26.6	25.5–27.8	**0.002**
No	13283	71.1	70.0–72.2	5454	28.9	27.8–30.0	
Can read and write							
Yes	24315	72.5	71.5–73.4	8879	27.5	26.6–28.5	0.610
No	7232	71.6	70.3–72.8	3128	28.4	27.2–29.7	
Race/color*							
White and yellow	14023	71.8	70.6–73.0	5205	28.2	27.0–29.4	0.125
Black, brown, and indigenous	17519	73.0	72.0–74.0	6801	27.0	26.0–28.0	
Per capita monthly income**							
≤1 minimum wage	14473	71.5	70.4–72.7	6001	28.5	27.3–29.6	0.069
>1 minimum wage	17074	73.0	71.8–74.1	6006	27.0	25.9–28.2	

95%CI: 95% confidence interval; *missing=6; **1 minimum wage in 2019 was equivalent to R$ 998.00 (Brazilian Reais). Statistically significant values are indicated in bold. Source: National Health Survey 2019.

In the analysis of the prevalence of influenza immunization in the elderly population who reported having some CNCD, significant differences were found in the groups with arterial hypertension 75.2% (p<0.001), diabetes 77.2% (p<0.001), and arthritis or rheumatism 75.5% (p<0.001) (Table 2).

**Table 2:** Prevalence of influenza vaccination in elderly Brazilians, according to chronic diseases.

Variables	Disease prevalence		Vaccination prevalence		
	%	95%CI	%	95%CI	p-value
Arterial hypertension	56.2	55.1–57.2	75.2	73.9–76.4	**0.000**
Diabetes mellitus	20.6	19.7–21.5	77.2	75.1–79.2	**0.000**
Arthritis or rheumatism	18.0	17.2–18.8	75.8	73.5–77.9	**0.000**
Heart disease	13.2	12.5–13.9	71.8	69.1–74.4	0.960
Cancer	6.9	6.3–7.5	71.5	64.8–77.5	0.185
Stroke	5.4	5.0–5.8	74.9	70.7–78.6	0.118
Asthma	4.7	4.3–5.2	75.5	71.1–79.3	0.078
Lung disease or COPD	3.0	2.7–3.4	75.5	69.0–80.9	0.231
Chronic kidney failure	2.5	2.2–2.8	71.5	64.8–75.5	0.944

COPD: chronic obstructive pulmonary disease; 95%CI: 95% confidence interval. Statistically significant values are indicated in bold. Source: National Health Survey 2019.

There was not a statistically significant difference in the prevalence of vaccination among the groups of elderly individuals diagnosed with heart disease (71.8%), cancer (71.5%), stroke (74.9%), lung disease or COPD (75.5%), asthma (75.5%), and renal failure (71.5%) (p>0.05) (Table 2).

The analysis of the prevalence of chronic disease immunization revealed significant differences (p<0.05) in the prevalence of vaccination for arterial hypertension, diabetes mellitus, and arthritis or rheumatism.

**Figure 2:**
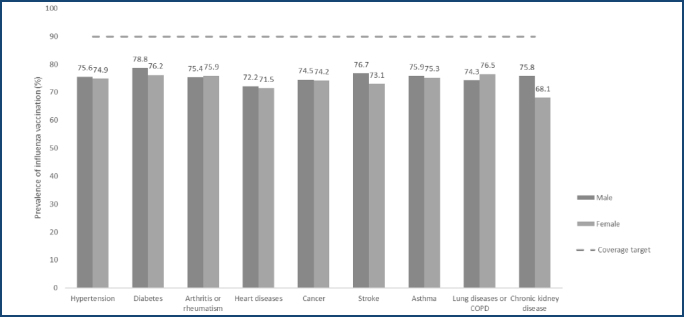
Prevalence of influenza vaccination in elderly Brazilians with chronic diseases by gender. COPD: chronic obstructive pulmonary disease. Source: National Health Survey 2019.

Greater adherence to immunization, according to gender, was observed in males, with hypertension and diabetes, and in females, with arthritis or rheumatism (Figure 2).

## DISCUSSION

This study analyzed data from the PNS in Brazil to assess the prevalence of influenza vaccination among the elderly population. The PNS is considered the main health survey in Brazil, providing population estimates that serve as a reference for other surveys and for monitoring basic health indicators among Brazilians^12^.

The results showed that the prevalence of influenza vaccination among the elderly population was low in 2019, with the estimates below the target of 90% established by the Ministry of Health for all evaluated groups with chronic noncommunicable diseases (CNCDs), indicating significant under-immunization^8^. This finding is consistent with a study by Bacurau and Francisco in 2013, which also found that the goal of coverage against influenza in elderly people with chronic diseases outlined by the ministerial body had not been achieved except for a single condition^8^.

The main reasons for low vaccination rates reported in the literature include rarely getting the disease, fear of adverse reactions, lack of trust in vaccine efficacy, and not knowing that vaccination is necessary^13,14^. This low adherence may be related to the phenomenon of vaccine hesitation, where individuals delay accepting or refusing recommended vaccines despite their availability in health services^15-17^. However, it becomes important to understand the reasons related to nonvaccination among elderly people with chronic diseases, even in the face of evidence showing a decrease in morbidity and mortality from influenza and a reduction in complications of chronic conditions. The ongoing monitoring of vaccination coverage in this specific population is a crucial condition for clarifying the reasons and analyzing the national trend in vaccination coverage.

Regarding sociodemographic characteristics, the study found that elderly females had a higher prevalence of vaccination compared to their male counterparts, which is consistent with previous studies^8,13,14,18-21^. However, a study carried out in Campinas-SP found a higher prevalence of vaccination against influenza in men^9^. The study also found that older elderly people had higher vaccination coverage than younger people, likely due to their greater vulnerability and health conditions that generate concern and result in greater adherence to vaccination^19^.

The study identified a higher prevalence of influenza vaccination among elderly people who live at home with a spouse or partner, a finding that supports the association identified by a previous Brazilian study linking living alone with incomplete vaccination status among elderly people^19^. This suggests that elderly people with social support are more likely to prioritize their health care.

Finally, the study found a positive association between influenza vaccination and the diagnosis of hypertension, diabetes, and arthritis or rheumatism, which is consistent with previous research^21-23^.

Although the study has limitations, such as the inability to establish a causal relationship due to its cross-sectional design and reliance on self-reported information, its results are generalizable for national estimates, given that it was conducted with a representative sample of elderly Brazilians. Furthermore, the PNS is a highly methodologically rigorous survey that allows for adjustments for potential confounding factors. However, it is important to note that the study’s estimates may be subject to information bias due to memory issues, lack of knowledge, or lack of a formal diagnosis of some health conditions.

Furthermore, to the best of our knowledge, there are no recent publications that have estimated the national coverage of influenza immunization among the elderly, and the last study in this area was based on data from the 2013 PNS, leaving a significant temporal gap. Therefore, our work fills a gap in the specialized literature by providing updated and representative data on national reality in 2019, the year in which the PNS was conducted. This contributes to the understanding of the current state of influenza immunization in this population group.

This study does not exhaust the subject, and additional studies should be conducted to assess the temporal trend of vaccination coverage and to investigate the associated factors.

## CONCLUSION

A global prevalence of influenza vaccination of 72.4% was estimated among elderly people in Brazil. This study highlights that influenza vaccination rates among the elderly population in Brazil in 2019 fell below the target set by the Ministry of Health.

It is crucial to promote vaccination against influenza in this age group, especially among individuals with chronic diseases, to mitigate the risk of complications, hospitalizations, and deaths associated with the virus. To achieve this, the government and healthcare organizations must implement strategies that aim to increase vaccine uptake and awareness, such as public education campaigns, improved accessibility to vaccines, and targeted outreach to vulnerable populations.
